# Study on the Formation of Novel Threadlike Structure through Intravenous Injection of Heparin in Rats and Refined Observation in Minipigs

**DOI:** 10.1155/2013/731518

**Published:** 2013-06-26

**Authors:** Yu-Ying Tian, Xiang-Hong Jing, Shun-Gen Guo, Shu-Yong Jia, Yu-Qing Zhang, Wen-Ting Zhou, Tao Huang, Wei-Bo Zhang

**Affiliations:** ^1^Institute of Acupuncture and Moxibustion, China Academy of Chinese Medical Sciences, Beijing 100700, China; ^2^Beijing University of Chinese Medicine, Beijing 100029, China

## Abstract

*Objective*. To study if the novel threadlike structure (NTS) was caused by coagulation during injecting urethane intraperitoneally and the source of NTS. *Methods*. Twenty-two SD rats were anaesthetized by urethane injected intraperitoneally. Heparin was injected at 5 minutes before the anaesthesia from femoral vein in 11 rats, and saline was given in the other 11 rats randomly. Six Chinese minipigs were carried to look for NTS. One sample was taken to be stained by DAPI/Phalloidin and observed by a laser scanning confocal microscope. *Results*. In the group of heparin, 10 rats were found to have NTS with appearance rate of 90.9%, and 9 rats were found to have NTS with the appearance rate of 80.1%. Both groups have 1.81 average numbers of NTS in each rat without significant difference (*P* > 0.05). In the observation of pigs, the NTS was found to prolong from the serous membranes of abdominal wall and organ surface. Histological observation showed elongated nuclei and alignment which is similar to the characteristics of PVS. *Conclusion*. There is no strong evidence to say that the NTS on organ surface was caused by coagulation of blood. The source of NTS might be a prolonged structure from serous membrane in abdominal cavity during the development and more or less retained after birth.

## 1. Introduction

Recently, a novel threadlike structure (NTS) was found on the surface of different organs in rabbits, mice, and rats by Korean team [1–4]. It was named primo-vessel system (PVS) by professor Soh as an extension of acupuncture meridians [5]. More recently, an experiment was carried by Choi and Leem that when injecting heparin intravenously at 5 min before the experiment, NTS could not be observed [6]. The rats were anaesthetized by Zoletil (50 mg/Kg) and xylazine (10 mg/kg). The author pointed out that when usually anaesthetizing rats by urethane injected intraperitoneally, it was easy to cause a slight damage of internal organs and bleeding in abdomen which may produce artifacts of NTS by coagulated string. But the author did not observe the appearance of NTS under the condition of urethane intraperitoneal anaesthesia and giving heparin before the operation. So, conclusion that NTS was caused by coagulated string when injecting urethane intraperitoneally was unclear. 

The target of this study is to investigate the formation of NTS. Two methods were used around the goal. One is that we tried to damage the structure by giving heparin under the condition of intraperitoneal anaesthesia of urethane, assuming that it is formed by a coagulation of blood. The second is that we observe the structure closely on minipigs to see the continuity with other tissues or organs under the condition of both intraperitoneal anaesthesia and intramuscular anaesthesia.

## 2. Materials and Methods

### 2.1. Heparin Experiment in Rats

Twenty-two Sprague Dawley (SD) rats (male, weight from 180 g to 376 g) were carried on the experiment. The rats were anaesthetized by intraperitoneal injection of urethane (1.5 g/kg). Then, they were randomly divided into two groups. In one group, 0.2 ~ 0.25 ml of heparin (5000 IU/mL, Sigma Co.) according to the weight was carefully injected from femoral vein, and then the place of injection was pressed for 5 minutes. In the control group, the same amount of saline was injected from femoral vein. After 5 minutes, the medial alba of the rat's abdomen was cut under deep anesthesia with the help of subcutaneous injection of small amount of xylocaine. The abdomen was then opened as much as possible to expose the internal organs. Bleeding was avoided by forcipressure. 0.4% Trypan blue solution (Sigma Co.) was obtained and was diluted to 0.1%. The 0.1% Trypan blue was further filtered through 0.22 *μ*m pore-sized filter paper just before the experiment. After exposure of the internal organs of the rats, Trypan blue was poured evenly on the exposed organs. After about one minute, the dye was washed away with 45°C warm saline, and NTS was searched through a stereomicroscope (Nikon SMZ750). If the NTS could be seen, the images were captured by the autocollection system in the stereomicroscope. The person who did the Trypan blue stain and observed the NTS was separated from the person who did the injection of heparine or saline which was a kind of blanking method.

### 2.2. Observation of NTS in Minipigs

The experiment of searching NTS was also done on six Chinese minipigs in different periods. Five pigs were anaesthetized by injecting 1.5 to 2 mg/kg of 2% phenobarbital sodium solution intraperitoneally. One pig was anaesthetized by injecting phenobarbital sodium solution (0.3 mg/kg) and xylazine hydrochloride injection (0.1 mg/kg) intramuscularly. Under deep anesthesia midline incision was carefully performed, and we passed through the following structures: skin, linea alba, transversalis fascia, extraperitoneal fat, and peritoneum. The incision was extended by cutting around the umbilicus while avoiding the falciform ligament above the umbilicus. Special care was also taken for the urinary bladder. Intra-abdominal organs were exposed carefully.

In the five minipigs which were anaesthetized by phenobarbital sodium intraperitoneally, 0.4% Trypan blue solution (Sigma) was diluted to 0.1% and filtered through 0.22-*μ*m pore-sized filter paper just before the experiment. After exposure of the internal organs, Trypan blue was applied on the exposed organs and was washed away with warm saline after about one minute. In the last minipig which was anaesthetized by phenobarbital sodium and xylazine hydrochloride injection intramuscularly, candidate NTS was searched directly without giving Trypan blue for getting the original color of NTS.

NTS was searched directly by eyes and was taken by a digital camera (Nikon D5000) with a 105 mm macro lens (Micro-Nikkor 105 mm f/2.8 G). A sample of candidate NTS from liver surface in the last minipig was harvested and fixed with 4% paraformaldehyde in 0.1 M phosphate buffered solution (PB, pH 7.4) for 2 hours at 4°C, then changed into 25% sucrose in 0.1 M PB (pH 7.4) for further examination. Serial longitudinal sections of NTS were cut at a thickness of 20 *μ*m on a cryostat (Thermo, Microm International FSE, Germany) and mounted on silane-coated glass slides.

For staining, the mounted NTS was incubated in a solution containing 3% normal goat serum and 0.5% Triton X-100 in 0.1 M phosphate buffered solution (PB, pH 7.4) for 30 min. After 3 times rinsing with 0.1 M PB, the sample was stained with Alexa Fluor 488 Phalloidin dissolution (1 : 50; Molecular Probes, Eugene, OR, USA) for 2 h, then washed with 0.1 M PB. After that, the sample was coverslipped with DAPI (Molecular Probes, Eugene, OR, USA). In this study, Alexa Fluor 488 Phalloidin and DAPI were used for identifying the morphology of F-actin and cell nuclei.

## 3. Results

### 3.1. Heparin Experiment in Rats

**Figure 1:**
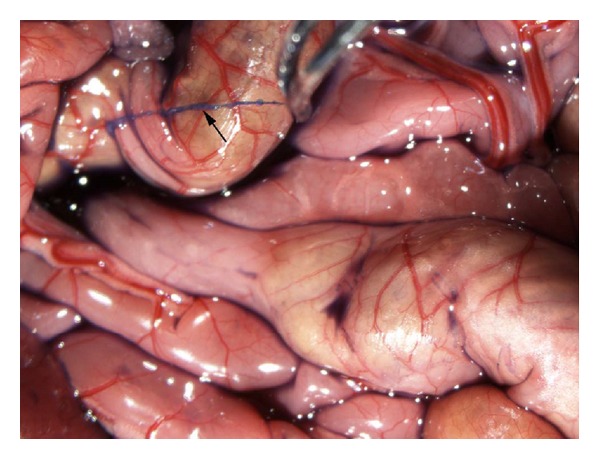
A blue color threadlike structure was found (↑) on the surface of intestine of a rat with intraperitoneal injection of urethane and giving heparin at 5 minutes before the incision.

**Figure 2:**
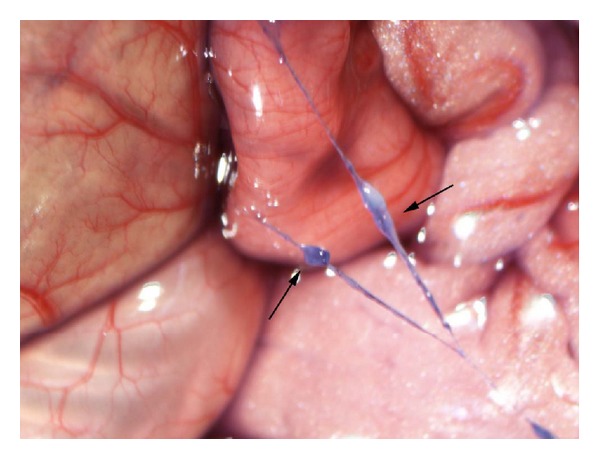
On enlarged sight of NTS, a node (↑) could be found on both two threadlike structures.

The two groups of rats have similar average weights which were 260.6 g (SD = 83.7) in heparin group and 289.7 g (SD = 79.4) in saline group without significant difference (*P* > 0.05). On the group of injecting urethane, among 11 rats, 10 rats were found to have NTS on the surface of intestine or liver.  Figure 1 showed one of them. The candidate NTS was blue color and can easily be separated from the surface of organ by a forceps. If we enlarged the sight by amplifying the picture, a node could be found clearly on most NTSs which was one of the criteria of discriminating NTS (Figure 2). On the control group of 11 rats, 9 rats were found to have NTS with the appearance rate of 81.8% which was similar to the appearance of heparin group. 

We also counted carefully the numbers of NTS on the surface of organs on each rat. It was found that the mean number of NTS on heparin group was 1.82 (SD = 0.87) and 1.82 (SD = 1.17) on control group. There was no significant difference between the two groups (*P* > 0.05). 

### 3.2. Observation of NTS in Minipigs

**Figure 3:**
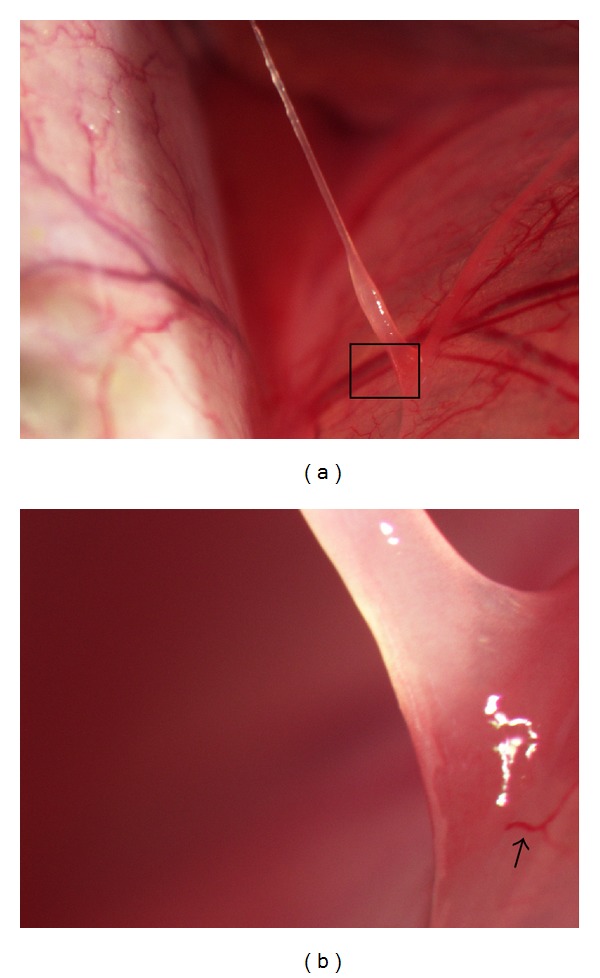
(a) A threadlike structure was found on the surface of stomach. (b) A further enlarged picture of (a) in pane to show the connection between the NTS and the surface of the organ. A small blood vessel with red color could be seen beside the NTS (↑).

For the observation on minipigs, the candidate NTSs on the organ surfaces like stomach, intestine, and liver were also found. In the five minipigs anaesthetized by phenobarbital sodium intraperitoneally, some samples were stained with DAPI, and rod-shaped nuclei were found [7]. A large sample on the surface of stomach was taken, and a further enlarged picture was obtained to see the refined structure rooted on the surface of stomach (Figures 3(a) and 3(b)).

The picture showed that the connection between the NTS and the surface of stomach was firmed and continuous from a restis on the surface of stomach. The NTS had nearly no color itself compared with the red color of small blood vessel nearby and appeared semitransparent.

**Figure 4:**
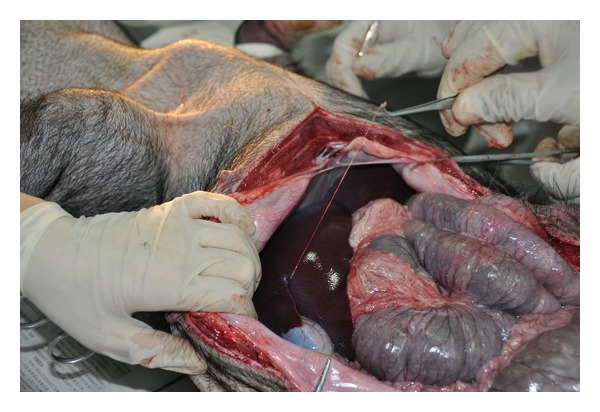
A threadlike structure was found on the surface of liver in one minipig with a tight connection with the liver.

**Figure 5:**
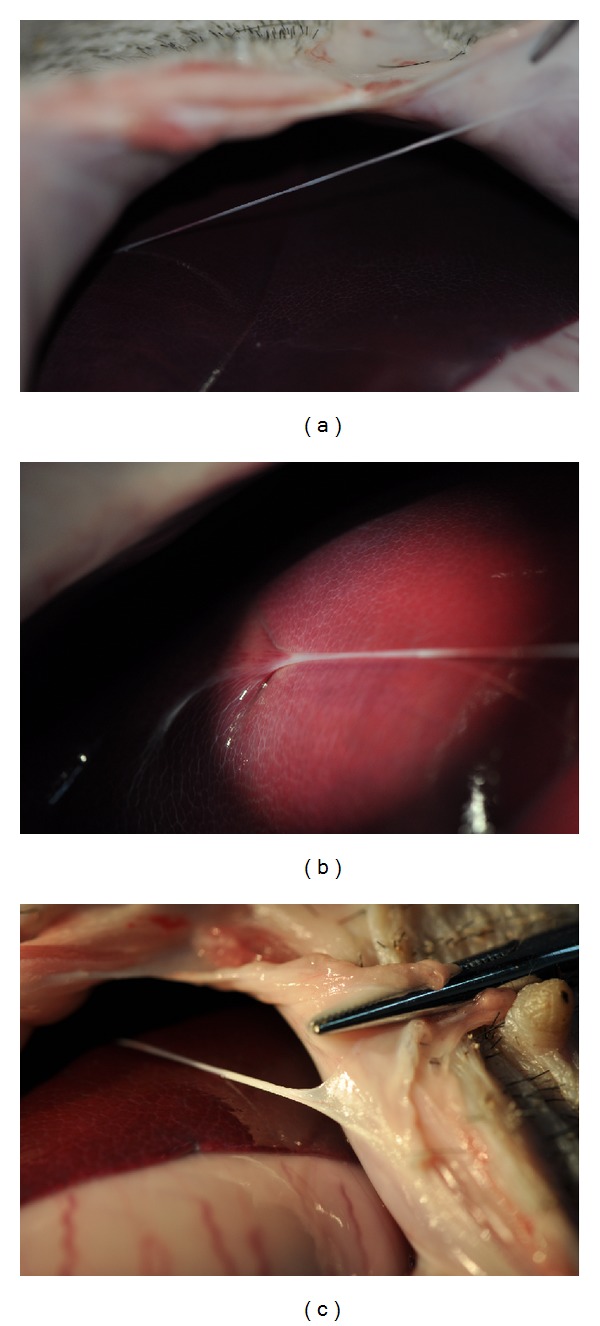
(a) A threadlike structure was found on the last minipig at a position similar to the one in Figure 4. (b) The root structure on the surface of liver. (c) The root structure on the abdominal wall.

In the other minipig, a thick NTS was found on the surface of liver connected to the abdominal wall (Figure 4). In the last minipig which was anaesthetized by phenobarbital sodium and xylazine hydrochloride injection intramuscularly, a candidate NTS was found at a position quite similar to the last one (Figures 5(a), 5(b), and 5(c)). The connections on the surface of liver and abdominal wall were tight, and the strings were elastic.

From Figures 5(b) and 5(c), prolongations of the threadlike structure from serous membrane on the abdominal wall and surface of liver could be seen clearly. 

## 4. Histological Observation

**Figure 6:**
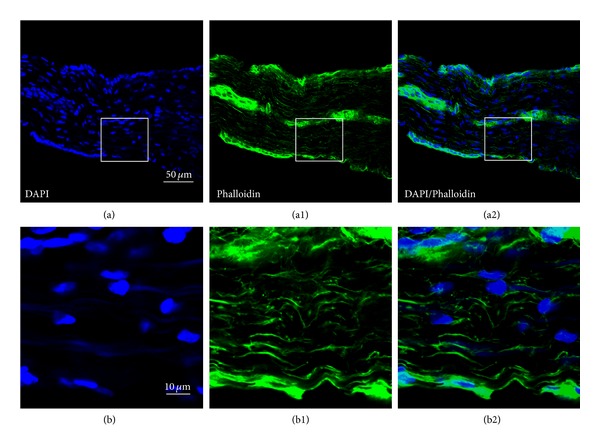
(a)–(a2) The structure of NTS in pig determined by fluorescent staining with DAPI (a) and Alexa Fluor 488 Phalloidin (a1) and their double labeling (a2). (b)–(b2) The magnified photos from the squares in (a)–(a2), respectively, showing the DAPI/Phalloidin-positive cells in detail. Scale bar, same for (a)–(a2) (showed in (a)) and same for (a)–(b2) (showed in (b)).

The tissue samples were observed and recorded with a laser scanning confocal microscope (FV1000, Olympus Co., Tokyo, Japan). Digital images were finally processed with Adobe Photoshop CS2 (Adobe Systems, San Jose, CA, USA). The result was shown in Figure 6.

From Figure 6, the cells labeled with DAPI/Phalloidin had elongated nuclei and flattened processes with alignment scene, parallel to the longitudinal direction of the sample which is similar to the characteristics of PVS and the features of fibroblasts. The distribution of nuclei and alignment is not even which showed higher density on the edge and central areas of the sample.

## 5. Discussion

A novel threadlike structure (NTS) was reported to be found on rabbits firstly by Bonghan Kim in North Korea in the early 1960s. As it is hard to be repeated by other scientists in other countries including China, the work had been stopped until KS Soh in South Korea claimed to find the similar structure again. The characteristics of NTS were studied extensively mainly concerning the structure, distribution, and relations with tumor, while the physiological function of NTS has not been studied intensively yet. We still do not know if it is a structure necessary for every living creature all the time or it is just a temporal structure that appeared during the development or at the pathological state. The formation of this structure is a secret. Some people thought that NST is just a coagulated string during anaesthesia or operation. An experiment was done by Liu in China to see the influence of heparin on the formation of NTS [8]. The rabbits were perfused by saline plus heparin or epinephrine through a femoral vein on one hindlimb and opened the femoral artery on the other hindlimb. It was found that NTS increased the length when epinephrine was given and disappeared when giving heparin. This is a good evidence to support that the NTS in blood vessel is formatted by a coagulation of blood. But outside the blood vessel, there is little chance to coagulate a string without serious bleeding. Our result showed an equal appearance of NTS on giving heparin and saline. So there is no strong evidence to say that NTS on organ surface is caused by coagulation. 

Choi and Leem did observe the influence of heparin but only on the rats when injecting Zoletil and xylazine intramuscularly. There was no condition to cause the coagulating string according to the author's view. The sample he used was small, and no blanking method was taken [6]. As searching NTS was carried out by man's observation, it is easy to have bias when knowing the condition. Our blanking method can avoid the bias from the person who observed the NTS. 

Also the damaged fractals from organ surface during the intraperitoneal anaesthesia are hard to form a string so quickly on the organ surface in a short time. Even if it was formed, it is impossible to form a tight connection with the internal organs or abdominal wall which has been found in minipigs. In the last minipig, intramuscular injection was used to avoid any damage on internal organs; the candidate NTS can still appear. 

For the result of minipigs, the much thicker samples than those of rats and rabbits could be observed easily at anatomical lever and made it possible to observe the refined structure of NTS. Some candidate NTSs do not freely stay on the surface of organs. They are continuous from the serous membrane of internal organs or abdominal wall which implied the origination of such structure. Histological result showed that a rod nuclei structure existed on the structure [7] which is coincident with criteria of PVS, and they can be stained by Trypan blue [7]. However, the sample from the last minipig had more bundles of cells which is similar to the observation by An et al. on no-free NTS in mesentery of rats [9]. The NTS we found in minipigs which had a tight connection with organ surfaces or abdominal wall is the same type of NTS found by Ping An et al. in mesentery of rats and was recognized as PVS [9].

Although not all cellular components in NTS could be determined by DAPI/Phalloidin labeling, our results suggested that fibroblasts might be an important cellular component in the NTS of pigs which implied the similarity of NTS with the loose connective tissue or fascia. 

The criteria of discriminating NTS as PVS were obtained on four aspects which were node and threadlike structure, bundle structure, rod shaped nuclei, and Trypan blue stainability [6]. As the limitation of the condition, we did not check the rod nuclei and bundle structure on all the samples. But a node threadlike structure and Trypan blue stainability are quite clear. Trypan blue can stain dead cells and is often used to discriminate the cells of living or death. The other dye, Alcian blue, which is also sensitive to NTS is often used on evaluating the density of mucopolysaccharide. Both dyes could not stain the living cells. So, a conjecture was obtained that NTS might originate from serous membrane between the abdominal wall and surface of organs during the development. There is no complete separation of serous membrane between the abdominal layer and organ layer or between the organs. Such connections might have certain functions of transporting nutrition and information from the mother through umbilical cord during the embryonic period and are more or less kept after birth, while the function is mainly replaced by self-circulatory and neural systems. This retained structure could be separated under certain unknown condition to become free style of NTS. This speculation could be tested by embryo experiment to trace the origin of NTS.

Lee et al. discussed the difference between free style and no-free style PVS and thought that they are two different tissues; namely, the no-free NTS is not a primo vessel [10]. It is quite difficult to distinguish the two things as both have rod-shaped nuclei and can be stained by Trypan blue. The differences are that free PVS has branches, node, and clear bundle, while no-free NTS has no (or less) branch, node, and clear bundle. But these differences are relative. A node may produce along a thread structure when the two ends of the thread become weak or released. For the similar reason, a bundle inside the thread may become unclear (broken) if the thread is strained and the branches may be damaged. Considering the communicating function of the tissue, a tight connection is better than loose connection on running the communication. However, two types of NTS might be the same or quite similar tissues. Study at molecular level should be done to discriminate the two styles of NTS.

## 6. Conclusion

NTS on internal organ surface is not formed by a coagulation of blood or damaged fractals from organ surface, while it has a close relationship with the serous membrane on abdominal cavity and organ surfaces.

## Supplementary Material

Verification on the entity of meridians reported by Bonghan Kim.
