# Supplementary material for: Study on the Formation of Novel Threadlike Structure through Intravenous Injection of Heparin in Rats and Refined Observation in Minipigs
Source: Evid Based Complement Alternat Med. 2013 Jun 26;2013:731518. doi: 10.1155/2013/731518 (PMC3710608; doi:10.1155/2013/731518)
Supplement: Supplementary file 1 — Verification on the entity of meridians reported by Bonghan Kim. [file 731518.f1.pdf]

☆短篇报道☆

关于“金凤汉经络实体”的验证工作

刘 乡
(中国中医科学院针灸研究所, 北京 100700)

【关键词】 经络实体 金凤汉 验证
【中图分类号】R 224.1 【文献标志码】B 【文章编号】1000-0607(2009)05-0353-02

中医和针灸疗法起源于中国, 经络学说是中医解释人体的生理机能、病理机制、临床症状的重要理论。但我国几十年来的研究均未找到独立于神经、血管和淋巴之外的经络系统。1961 年, 朝鲜金凤汉发现机体内存在有经络的实体结构, 穴位内存在“凤汉小体”, 血管内存在有“凤汉管”。我国对朝鲜的重大科学发现非常重视, 1963 年在《人民日报》《光明日报》以数版篇幅全文刊载了金凤汉“关于经络系统”的学术论文。随后, 我国由一流生理学、组织学和病理生理学的专家组成考察团, 赴朝到金凤汉研究室参观学习, 回国后由中年科技人员, 在北京、上海、沈阳和长春等地分别成立了研究小组。全国各研究组按金凤汉的方法以及能想到的各种方法进行了研究和观察, 但均未找到令人信服的经络实体结构。后因种种原因, 此项研究不了了之。

不想沉寂 40 年后, 金凤汉经络实体的研究又被重新提起, 并有新的研究证明它的存在。韩国苏光燮认为“金凤汉的经络系统”是半透明纤细的, 因而用肉眼和手术显微镜难以看到。2002 年他们的研究小组用生物影像学和生物标记等新技术, 找到了“金凤汉的经络系统”[1]。旅日学者李强甚至建议: 在以后出版的经络书中, 必须给金凤汉平反[1]。但他们的研究结果与金凤汉的研究内容是否一致尚不得知, 因为金凤汉发现的“凤汉小体”和“凤汉管”并不纤细, 而且他当时也未应用什么特殊先进的技术。

遗憾的是, 我国验证工作的详细资料未公诸于世, 只得任凭他人评说。当年的老科学家早已仙逝, 当时的中年人现都已届耄耋之年, 有些人也已远行。我辈尚存者有责任把记忆中的资料记述出来, 否则我国投入大量人力物力, 历时 3 年多的研究岂不白白浪费! 为此, 笔者愿以负责的态度, 把当年亲自进行的验证工作所得的结果, 以及自己所知道的有关情况尽量详细地回忆记述出来, 供有关部门和研究工作者参考。

当时吉林医科大学(白求恩医科大学)从生理、生化、解剖、组织和病理等科室抽调了 6 人, 建立形态和机能两个研究组, 从 1963 年到 1966 年进行的验证工作如下。

一、形态组 4 人(石世庆、代桂林、李博和许以侣), 每日按金凤汉的方法, 在解剖镜观察下, 用镊子小心拔去家兔后肢根部内侧或“足三里”处兔毛, 寻找约 1 mm 大小的“凤汉小体”; 还在兔、小牛, 甚至刚死亡人的足三里部取皮肤, 做连续切片, 均未找到“凤汉小体”。后来, 北京医学院李肇特教授和沈阳医学院的杨枫教授在幼兔的肚脐内找到与“凤汉小体”类似的结构, 为内含嗜铬组织、外包有平滑肌的小体。但家兔长到成年以后此小体消失, 证明此乃脐带退化的组织结构。

二、生理组两人(刘乡和杨翰仪), 按金凤汉的方法记录“凤汉小体”电位。从家兔“足三里”部位取出皮块, 固定于盛台氏液的湿室内, 即一长、宽、高各约 5~6 cm 的有机玻璃盒, 置于 37 ℃ 恒温水浴内保温, 以针灸针做为电极, 与导线焊接连至阴极线示波器。当两根针灸针电极插入皮块内时, 在示波器上可记录到波形、速度都与“凤汉小体”电位一致的电波。但经我们进一步分析, “小体电位”与两个电极插入台氏液中的深度有关: 如两针灸针电极在台氏液内处在同一深度则电位消失; 如一个电极仅不锈钢针部分插入台氏液中, 而另一电极插入液面较深在银丝(缠绕针灸针柄)或铜线水平, 则“小体电位”出现, 即使将皮块取出, 电位的波动仍然存在。因此可以证明, 此“小体电位”并非生物电, 而是不同金属间的电位差所造成的。这项研究的结果明确一致。

三、我们按金凤汉灌流的方法, 于家兔一侧后肢股静脉插管连接生理盐水或台氏液吊瓶, 而另一侧

© 1994-2012 China Academic Journal Electronic Publishing House. All rights reserved. http://www.cnki.net

后肢股动脉插一塑料管, 为放血之用。随着灌流放血, 家兔的血液逐步被灌流液体所取代, 流出的血液逐渐变淡, 最后完全变成白色的灌流液, 家兔因失血而死亡。此时切开胸腔和腹腔, 暴露出心脏和主动脉, 小心剪开胸主动脉、腹主动脉及其动脉分支, 在胸主动脉可看到一白色索状物, 通常可延续到腹主动脉。笔者曾持此物到北京向赴朝的教授请教, 回答与他亲眼所见到的"凤汉小管"一致。因此我们成功地重复出金凤汉的血管内"凤汉管"。但我们进一步的实验证明, 如在灌流液中加入促进血液凝固的肾上腺素, 则所形成的索状物, 不仅存在于胸主动脉内, 而且也可连续出现在腹主动脉及其分支动脉中, 逐渐变细而于远端消失; 若在灌流液中加抗血凝的药物肝素, 则灌流后动物血管中此索状物不再出现, 屡试不爽。表明金凤汉所发现的血管内"凤汉管"可能是与血液凝固有关的纤维蛋白束。那么什么样的先进技术能标记或染出一个在正常生理状态下并不存在的结构呢? 我们进一步对此索状物进行染色, 有时能观察到零散的上皮细胞, 这可能是用镊子夹取时, 碰到血管壁带到索状物上去的, 但并未看到上皮细胞连续构成的管状结构。

四、我们还在鄂征教授制作的兔皮管诱生窗[2]中观察到活体新生血管内血液流动中似有管状的影像。如给动物静脉注入蓝色染料(似为台盼蓝), 则能清楚见到蓝色的细流在血管中间流动, 更似细管。经模拟实验证明其并非真正的管状结构, 而是液体在管道中流动时的轴流现象, 即液体靠近管壁侧由于摩擦阻力大流速减慢, 而中心部阻力小流速快, 因而在显微镜下观察到似管状影像。另外从生理功能思考, 动脉中悬着一根细管, 它能否经得住高达 100~200 mmHg 动脉血压的冲击? 如果细管脱落则会形成梗塞, 造成血液循环障碍, 危及生命。正常机体怎能有此种危害生命的结构存在, 岂不违反生物学意义及生物进化的规律!

《灵枢·经脉》已对经络的结构进行过描述: "经脉十二者, 伏行于分肉之间, 深而不见。""诸脉之浮而常见者皆络脉也。"其所说的"经脉"相当于走行于四肢肌肉之间的动静脉管和神经干, 而"络脉"相当于皮下的静脉和毛细血管。《灵枢》指出经络的功能, 内属于脏腑, 外络于肢节, 沟通内外, 贯通上下, 将人体各部组织和器官联系成为一个有机整体; 并借以运行气血, 营养全身, 使人体各部功能得以保持协调和相对平衡[3]。古人对经络机能的阐述相当于现代医学的血液循环、神经控制和神经-体液调节等功能。

上世纪六、七十年代, 我国因"针刺麻醉"而开展了大规模的针刺镇痛机制研究, 认为疼痛的感受与调制是神经系统的功能; 生理学家即从神经生理学入手进行研究, 得出针刺穴位的传入途径是神经的结论[4]。针刺的镇痛效应是来自穴位和来自痛区的传入冲动在中枢神经系统内相互分析综合作用的结果[5]17-23; 外周传入信号沿脊髓腹外侧索上行, 可主要激活脑内包括中缝后核群在内的内侧网状结构, 以及某些高位脑结构综合作用, 然后沿脊髓背外侧索下行在脊髓水平, 抑制较细传入纤维(Aδ、C纤维)冲动的向上传递, 产生镇痛作用[5]24-29,[6]; 内源性阿片肽抗伤害性系统可被针刺激活并参与针刺镇痛的过程[7]。杜焕基对针刺的外周传入、中枢整合和反馈控制进行了全面的综述认为, 我国是当时世界上能对针刺镇痛的神经生理学机制进行比较全面、合理解释的唯一国家[8]。关于经络结构的研究至今仍无突破性进展, 笔者认为经络系统是古代医家从治疗经验中总结和推理出的人体调控和营养系统, 他们已观察到的结构就是血管、神经。我们应该遵循古代医家的思路, 使经络系统具体化和科学化, 溶入现代生理学和医学, 而不应将经络神秘化, 以免研究走入歧途。

参考文献

[1] 李江慧, 朱　兵. 又现金凤汉[C]. 北京: 中国中医科学院针灸研究所, 2008.

[2] 鲍鉴清. 组织学技术[M]. 长春: 吉林人民出版社, 1962: 77-90.

[3] 上海中医学院. 针灸学[M]. 北京: 人民卫生出版社 1974: 3-22.

[4] 江振裕. 针刺镇痛效应外周传入途径的分析[J]. 中国科学, 1973, 2: 157-161.

[5] 张香桐, 季钟朴, 黄家驷. 针灸针麻研究[M]. 北京: 科学出版社, 1986.

[6] 刘　乡. 以痛制痛——针刺镇痛的基本神经机制[J]. 科学通报, 2001, 46(7): 609-616.

[7] He L F. Involvement of endogenous opioid peptides in acupuncture analgesia[J]. Pain, 1987, 31(1): 99-121.

[8] 曹天钦, 冯德培, 张香桐, 等. 神经科学前沿[M]. 北京: 知识出版社, 1986: 190-217.

(收稿日期: 2009-06-15　修回日期: 2009-07-10)

© 1994-2012 China Academic Journal Electronic Publishing House. All rights reserved.　http://www.cnki.net
